# Rectal cancer developing from an anastomotic site 18 years after resection due to intussusception caused by Peutz-Jeghers polyposis in a 31-year-old man: a case report

**DOI:** 10.1186/s40792-018-0519-z

**Published:** 2018-09-05

**Authors:** Yuya Nobori, Takahiro Amano, Mieko Ochi, Toshio Kumasaka, Eiji Sunami

**Affiliations:** 1 Department of Coloproctological Surgery, Japanese Red Cross Medical Center, 4-1-22, Hiro-o, Shibuya-ku, Tokyo, 150-8935 Japan; 2 Department of Pathology, Japanese Red Cross Medical Center, 4-1-22, Hiro-o, Shibuya-ku, Tokyo, 150-8935 Japan

**Keywords:** Peutz-Jeghers syndrome, Rectal cancer, Anastomotic site, Heterotopic ossification

## Abstract

**Background:**

Peutz-Jeghers syndrome (PJS) is an autosomal dominant disorder characterized by hamartomatous polyposis of the gastrointestinal tract. It is associated with a high risk of malignancy in the gastrointestinal tract, as well as in other organs. We report a case of colon cancer at the anastomotic site that occurred 18 years after high anterior resection of the rectum for intussusception caused by Peutz-Jeghers polyposis.

**Case presentation:**

A 31-year-old man with PJS, who had undergone high anterior resection of the rectum for intussusception at the age of 12, presented to our hospital complaining of hematochezia. Colonoscopy revealed a hemorrhagic tumor protruding from the anastomotic site, which was histologically diagnosed as an adenocarcinoma. We performed a low anterior resection of the rectum including the anastomotic site, with combined resection of the strongly adherent ileum. Histological examination revealed that the adenocarcinoma had developed from the submucosal area, where the normal rectal mucosa had been incorporated into the stromal and bone tissues, resulting in heterotopic ossification in the anastomotic region. These findings suggested that the reconstructive surgical procedure or postoperative complications, such as anastomotic leakage, had formed the cavity where the cancer had developed.

**Conclusions:**

We concluded that the cancer might be derived from the rectal mucosa with malignant potential that was present in the anastomotic region and exacerbated by the presence of chronic inflammation in the cavity after the patient’s initial operation.

## Background

Peutz-Jeghers syndrome (PJS) is an autosomal dominant disorder characterized by hamartomatous polyposis and mucocutaneous pigmentation [1]. Patients with PJS are at risk of developing malignant tumors in the gastrointestinal tract and/or other organs [2], although the mechanisms that lead to tumorigenesis in the gastrointestinal tract are unclear. The cumulative risk of colorectal cancer in patients with PJS between the ages of 15 and 64 years is 39%, and the relative risk is 84, resulting in an average age of 43 years in affected patients [2, 3]. Local recurrence after resection often occurs in malignant gastrointestinal cancers [4] but is rare in patients with benign tumors or other conditions, except in some cases of ulcerative colitis [5, 6]. Here, we report a case of rectal cancer in a young man with PJS that arose from the submucosal area of the anastomotic site of a prior high anterior resection of the rectum, which had been performed 18 years ago due to benign hamartomatous polyposis.

## Case presentation

**Fig. 1:**
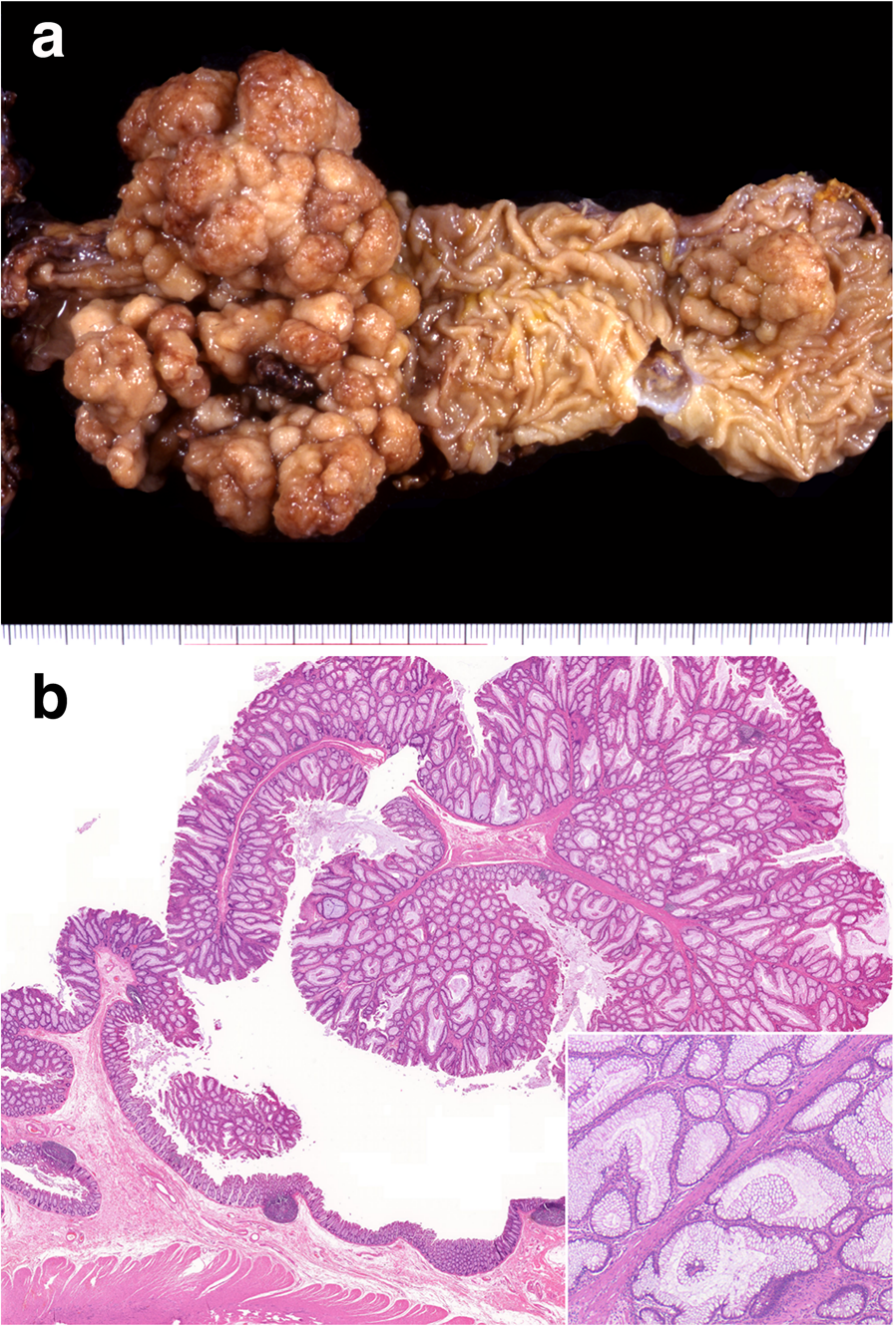
Peutz-Jeghers polyposis of the colon resected at the age of 12. **a** Macroscopic photograph showing the resected colon with a giant papillary polyp filling the lumen and some smaller polyps. **b** Microscopically, the polyps were multilobulated with branching bands of the muscularis mucosae covered by hyperplastic glandular mucosa (× 1; inset: × 10, hematoxylin-eosin stain)

**Fig. 2:**
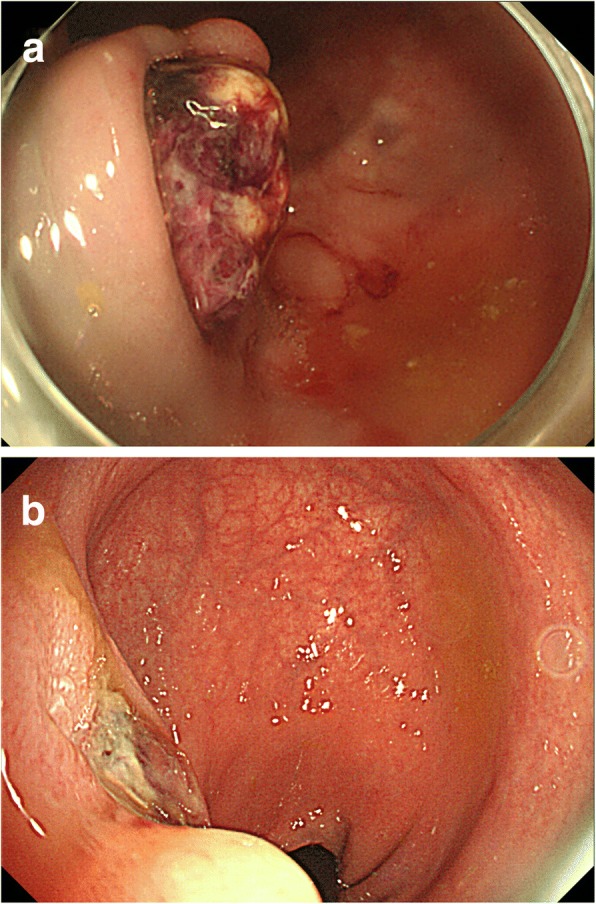
Endoscope images of the rectum at **a** the first examination and **b** at the second examination conducted 4 days later

A 31-year-old man was admitted to our hospital, complaining of hematochezia which had lasted for 1 month. His past history involved a high anterior resection of the rectum in our hospital due to intussusception caused by Peutz-Jeghers polyposis (Fig. 1). He was hospitalized for 1 month after the procedure. Unfortunately, details of the surgical procedure that had been performed and the reason for his extended postoperative hospitalization were unknown, because clinical records from his previous admission were not available. He was followed up for 3 years after the procedure, during which he had no abdominal symptoms. He remained symptom-free until the month prior to readmission when he began to suffer from hematochezia. On this admission, hematochezia was his only symptom and there were no abnormal abdominal findings on physical examination. Blood test results, including levels of tumor markers, were all within normal limits. Colonoscopy revealed a hemorrhagic tumor with a smooth surface protruding from the anastomosis of the previous high anterior resection, at a distance of 10 cm from the anal verge; a second examination 4 days later revealed that the tumor had disappeared (Fig. 2). The biopsied tumor and other small polyps were histologically diagnosed as adenocarcinoma and hamartomatous polyps, respectively (data not shown). We performed a low anterior resection of the rectum, including the anastomotic site with the adenocarcinoma, combined with a resection of the ileum for strong adhesion. The patient was discharged from our hospital 42 days after the operation.

**Fig. 3:**
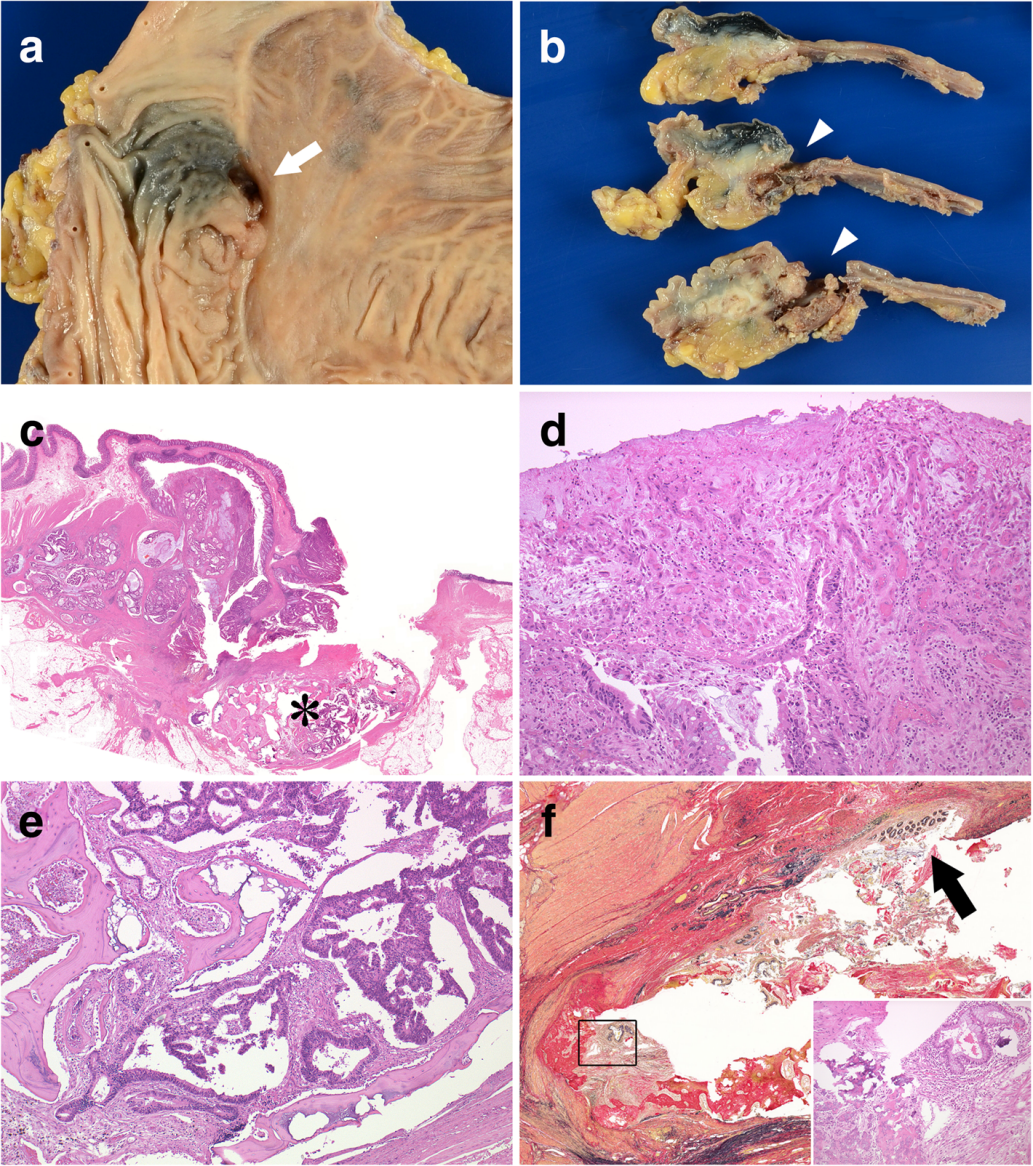
Macroscopic and microscopic photographs of the resected rectum. **a** The surface of the resected rectum shows mucosal defect (white arrow) and a submucosal tumor on the anastomotic line. **b** The cut surfaces of the rectum demonstrate a submucosal tumor with bone lesion underneath the mucosal defect (white arrowheads). **c** Histologically, the tumor with bone lesion (*) occupies the muscle layer and submucosal area (whole-mount image; hematoxylin-eosin [HE] stain). **d** Part of the surface of the tumor is covered by granulation tissue, indicating erosion (× 20; HE stain). **e** Image showing adenocarcinoma invading into the bone lesion (× 5; HE stain). **f** Normal mucosa in the submucosal fibrosis (black arrow) and the bone lesion in the proper muscle layer (black square) at the anastomotic site (× 2; Elastica van Gieson stain). Inset: a high-power view of the area within the black square (HE stain)

Gross appearance of the resected rectum showed a defect of the rectal mucosa with a smooth edge and a mucosal bulge located at the anastomotic site (Fig. 3a). Cut surfaces demonstrated a submucosal tumor mainly occupying the proper muscle layer under the defect (Fig. 3b). Microscopically, the submucosal tumor comprised an adenocarcinoma and a bone lesion at the anastomotic site (Fig. 3c). The surface of the tumor was covered with granulation tissue (Fig. 3d). The bone lesion not only included the carcinomatous glands but also normal glands in the bone tissue (Fig. 3e, f). In addition, we identified the incorporation of the normal mucosa in the submucosal fibrosis at the anastomotic site (Fig. 3f).

## Conclusions

In the present case, an adenocarcinoma arose from the submucosal layer of the anastomotic site of a previous high anterior resection for polyposis caused by PJS polyposis in a young man, 18 years after surgery. The cancer arising from the submucosal area was identified by both endoscopic and histologic examinations. Two preoperative endoscopic examinations were performed. The first revealed a hemorrhagic tumor with a smooth surface and edge protruding from the surface of the rectum, which appeared through the orifice of the rectal mucosa. Curiously, the second examination 4 days later showed that the protruding tumor had disappeared, which is rarely observed in common rectal cancers. We hypothesize that the neck of the tumor at the rectal orifice may have been disrupted since the patient later informed us that he had excreted small hemorrhagic masses before the second examination. Histological examination revealed that the cancer mainly occupied the proper muscle layer and that granulation tissue covered the surface of the tumor but not the surrounding rectal mucosa; this indicated that a disruption had occurred at the neck of the tumor, as it was free from the surrounding rectal mucosa.

In addition, histological examination demonstrated that normal rectal mucosa was incorporated into the bone and surrounding stromal tissues, both of which the cancer had invaded into. This suggests that rectal mucosa in the bone or surrounding stromal tissues exists prior to bone formation or cancer development since normal rectal mucosa cannot naturally invade into these tissues.

**Fig. 4:**
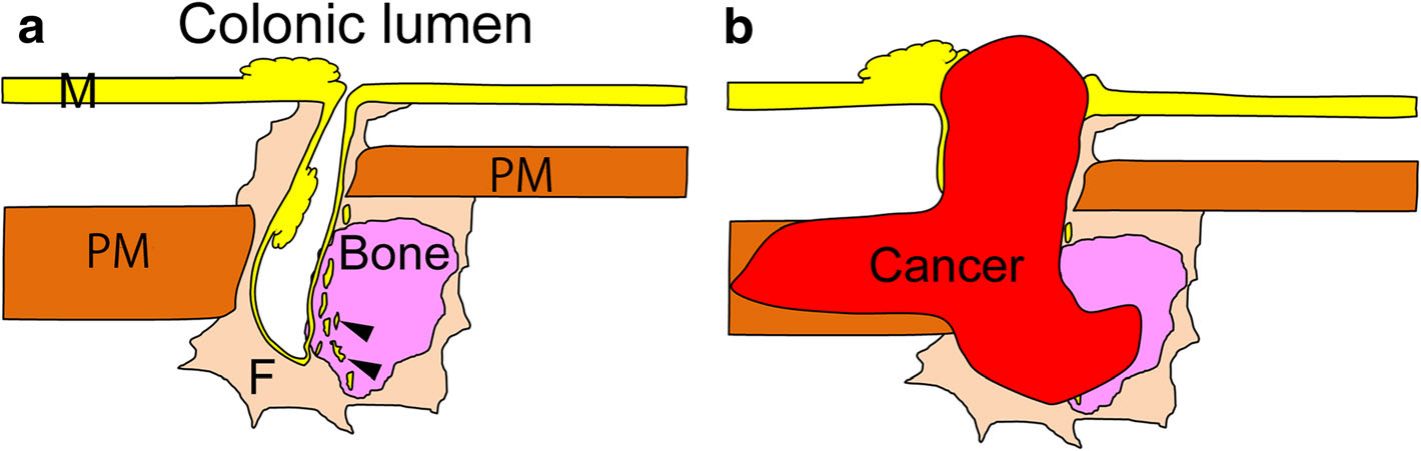
Scheme of rectal cancer developing from the anastomotic site in the present case. **a** Healing state of anastomotic leakage when the patient was 12 years old. The healing process of abscess formation by anastomotic leakage may lead to heterotopic ossification and repair of rectal mucosa, inducing the incorporation of rectal mucosa into the bone tissue (M, rectal mucosa; PM, proper smooth muscle layer; F, fibrosis of anastomotic site; arrowheads indicate rectal mucosa incorporated into the bone tissue). **b** Development of cancer at the anastomotic site 18 years later. Cancer may arise from the rectal mucosa in the cavity or the stromal tissue, subsequently invade into adjacent tissues including bone tissue, and protrude into the rectal lumen through the orifice of the cavity

We suggest that bone formation at the anastomotic site, or heterotopic ossification, is important for understanding this condition. In fact, although a few cases of ossification have been reported in Peutz-Jeghers polyposis or colorectal cancer, ossification in the gastrointestinal tract is extremely rare [7, 8]. Since heterotopic ossification commonly occurs as a complication of trauma [9], the process of wound healing after traumatic injury at the anastomotic region may have induced heterotopic ossification, as well as the incorporation of rectal mucosa into the bone and surrounding stromal tissues in our patient. We had knowledge of the patient’s clinical history of long hospitalization after high anterior resection, in addition to the findings from the second low anterior resection of strong adhesion between the anastomotic region and the surrounding intestines. Together, these clinical and histological data imply that traumatic injury such as anastomotic leakage may have occurred in this region (Fig. 4). An alternative explanation is that the cavity, a so-called dog ear formation, which results from traditional double stapling anastomosis, was formed due to the original surgical procedure; however, the lack of clinical and surgical records makes it difficult to precisely ascertain its origins.

Although there is a high risk of colorectal cancer in patients with PJS, cancer developing at an anastomotic site has never been reported. In cases of common colorectal cancer, local recurrence at the anastomotic site after surgery often occurs within 3 years, but rarely after 5 years [4, 10]. However, in the present case, the initial tumors were not malignant but benign hamartomatous polyps, which were completely resected. Therefore, it is possible that the cancer in this case arose independently of the initial Peutz-Jeghers polyposis. On the other hand, given the high risk of colorectal cancer in patients with PJS, it is possible that the cavity at the anastomotic site was covered with rectal mucosa with malignant potential [3]. Chronic persistent inflammation may have occurred within the cavity which, in addition to the cavity being in an enclosed environment, may have led to cancer development. This is similar to what occurs in inflammatory bowel diseases such as ulcerative colitis that secondarily develop into colorectal cancer [11]. Thus, we concluded that the patient with PJS may have developed cancer in the rectal mucosa with malignant potential that was located in the submucosal cavity or had been incorporated into the bone or stromal tissues. We hypothesize that this was further stimulated by chronic persistent inflammation during the 18-year period after the initial high anterior resection of the rectum due to Peutz-Jeghers polyposis.

## Abbreviation

PJS: Peutz-Jeghers syndrome
